# Association Between Hypertensive Disorders of Pregnancy, Lifestyle Factors, and Premature Cardiovascular Events

**DOI:** 10.1016/j.jacadv.2026.103187

**Published:** 2026-08-31

**Authors:** Simone L. Marschner, Desi J. Quintans, Clara K. Chow

**Affiliations:** a Westmead Applied Research Centre, The University of Sydney, Westmead, New South Wales, Australia; b Department of Cardiology, Westmead Hospital, Westmead, New South Wales, Australia

**Keywords:** hypertensive disorders of pregnancy, lifestyle risk factors, premature cardiovascular event

## Abstract

**Background:**

Hypertensive disorders of pregnancy (HDP) increase the risk of future cardiovascular events.

**Objectives:**

The objective of the study was to examine the association between lifestyle factors and premature (<50 years) cardiovascular (pCV) events in women with and without HDP.

**Methods:**

Data from young parous women in the Australian Longitudinal Study on Women’s Health 1973-1978 cohort were linked with medical records. Time-varying Cox proportional hazards models were used to assess whether lifestyle factors were associated with pCV in those with and without HDP. Outcomes of pCV included stroke, ischemic heart disease, valvular heart disease, cardiomyopathy, select arrhythmias, valvular heart disease, pulmonary embolism, and deep vein thrombosis.

**Results:**

Among 10,368 parous women (median follow-up: 16.5 years), 14.4% (1,493/10,368) had HDP and 4.3% (448/10,310) experienced a pCV event. HDP (adjusted HR [aHR]: 1.74; 95% CI: 1.32-2.31), obesity (aHR: 1.60; 95% CI: 1.19-2.15), and high saturated-fat intake (>319 g/day) (aHR: 1.67; 95% CI: 1.11-2.53) were associated with an increased risk of pCV. Breastfeeding (>16 months) (aHR: 0.66; 95% CI: 0.49-0.89) and high protein intake (>90 g/day) (aHR: 0.65; 95% CI: 0.43-0.96) were associated with a lower risk of pCV. The association between smoking, the Australian Recommended Food Score diet, and glycemic index with pCV events differed in women with and without HDP.

**Conclusions:**

This study found that a HDP diagnosis was associated with pCV. Obesity and high saturated-fat intake were associated with increased pCV, whereas breastfeeding and high protein intake were associated with lower pCV. The impact of some risk factors on the development of pCV differs between women with and without HDP. These findings highlight the importance of prevention management in women with HDP.

## Background

Over 1 million Australian and 275 million women globally have heart disease,^1^ with cardiovascular (CV) disease (CVD) being the leading cause of death in women worldwide.^2^ In 2011, the United Nations set a target to reduce premature deaths from noncommunicable diseases including CVD by 25% by 2025 with most countries not meeting this target.^3^ The burden of CVD remains a persistent challenge. Hypertensive disorders of pregnancy (HDP) are common and include de novo gestational hypertension (GH) affecting 5% to 10% of pregnant women^4^ and preeclampsia (PE) and eclampsia (E) affecting 3% to 8% of pregnant women.^5^ HDP is associated with a 2-fold risk of developing CVD in women^6^, ^7^, ^8^ and a similar risk for premature (under 50 years) CV (pCV) events.^9^^,^^10^ One study of U.S. Medicaid data found young women (average age of 26 years) with a pregnancy-related cardiometabolic conditions had a 3-fold short-term risk (during pregnancy and 60 days postpartum) of severe CV outcomes.^9^ Another study of women of median age 30 years reported a 2-fold risk of premature CVD after long-term follow-up (average 4.6 years)^10^ Although HDPs are a well-recognized female-specific CVD risk factor, primary care studies indicate women with HDP have suboptimal rates of subsequent CVD risk screening and management.^11^^,^^12^

Lifestyle modification is commonly recommended to younger women to reduce CV risk; however, there are only a few small studies exploring lifestyle interventions, including nutrition and exercise education, and CV outcomes after a pregnancy with HDP.^13^ There are also fewer studies that focus on pCV events among young women. Further understanding of the relationship of HDP and pCV events will focus attention on this important women’s health predictive marker and inform ways to mitigate that risk.

Important behavioral risk factors associated with CVD are unhealthy diet, physical inactivity, tobacco, and alcohol use.^14^ The Dietary Approaches to Stop Hypertension (DASH) diet,^15^ the Mediterranean diet,^16^ the Ultra-Processed Food (UPF) diet,^17^ the Australian Dietary Guideline Index (DGI) diet^18^ and the Australian Recommended Food Score (ARFS) diet^19^ reduce risk of CVD. Similarly smoking,^20^ alcohol use,^21^ physical exercise,^22^ sleep,^23^ parity,^24^ and breastfeeding^25^ are lifestyle factors found to be associated with CVD risk. The Australian Longitudinal Study on Women’s Health (ALSWH) offers a large single cohort with frequent and detailed surveys on demographics, medical conditions and lifestyle. The aim of this analysis is to assess if lifestyle risk factors known to be associated with CVD outcomes mitigate the risk of pCV events after a HDP diagnosis.

## Methods

### Study design and population

This study is a longitudinal cohort study using linked data. The ALSWH is an Australian longitudinal survey of representative women from across Australia. In 1996, women were randomly selected from the Australian national Medicare database, which contains the name and address details of all Australian citizens and permanent residents, and sent an invitation to participate in the ALSWH. More than 40,000 responded and agreed to participate in the project for 20 years. Three age groups cohorts were created (born in 1946-1951, 1973-1987, 1989-1996) to follow women through life stages which are critical to women’s health and well-being.^26^^,^^27^ Surveys were administered every 3 years from July 18, 1996 – December 1, 1996 to June 23, 2021 to November 21, 2022. The study was approved by the Human Research Ethics Committees at the University of Newcastle and the University of Queensland, and informed consent was received from all ALSWH participants. The study is funded by the Australian Government Department of Health, Disability, and Ageing and managed by the University of Queensland and the University of Newcastle. In addition to the survey data, the ALSWH cohorts are linked to external administrative data collections. ALSWH participants who decline health record linkage are excluded from linked data requests. Over 80 percent of all ALSWH participants have explicitly consented to record linkage.

This study used parous women from the ALSWH 1973-1978 cohort to focus on women under 50 years and identify which lifestyle risk factors, known to mitigate the risk of CVD outcomes, are more effective in mitigating the risk for young women who have an elevated risk due to an HDP diagnosis. This study used the components in the ALSWH surveys on pregnancy, breastfeeding, diet, exercise, sleep and body mass index (BMI).

### Outcome

The primary outcome of this study is pCV events. pCV events included stroke, ischemic heart disease, valvular heart disease and cardiomyopathy, cardiac arrhythmias, valvular heart disease, and thrombotic disease including deep vein thrombosis as suggested by Joshy et al^28^ Although some of these events are less likely to be associated with lifestyle, we included them all as they are all important pCV events for young women. pCV events were any diagnosis of a major CV event (as listed previously) from the hospital records using International Classification of Disease-Australian Modified code identified as major CVD, supplemented by the “Common Conditions from Multiple Sources flags” of ischemic heart disease and stroke. Common Conditions from Multiple Sources flags were created by the ALSWH study team. They identified major health events through linked external health record collections including death records, Medicare benefits schedule, pharmaceutical benefits scheme, hospital admissions, emergency department, and aged care records together with their ALSWH surveys. The question on the ALSWH survey identifying pCV asked whether the doctor had told you that you have heart disease. There was a separate question on hypertension which was not included in this analysis. Women in this study cohort were aged 43 to 49 years at time of analysis. Therefore, any CVD events observed in this cohort, occurring before age 50, were considered pCV events.

### Exposure variable and covariates

The exposure variable HDP, made up of PE/E and GH, was sourced primarily from linked Perinatal Data Collections and further searched in the ALSWH surveys, hospital admissions, and emergency department data using International Classification of Disease - Australian Modified codes O11 and O13-O15. GH was asked in the ALSWH surveys referring to it as “Hypertension (high blood pressure) during pregnancy?”; however, PE/E were not recorded in the ALSWH surveys.

The lifestyle risk factors of interest were smoking status, alcohol use, sleep, physical activity, specified diets, and other diet variables such as total of each of the following: energy, carbohydrates, fat, saturated fat, polyunsaturated fats, protein, sugar, fiber, glycemic index and glycemic load. Circumstantial behaviors were breastfeeding and clinical characteristics were BMI and parity. Socioeconomic factors were also explored, namely remoteness, education, and country of birth. Alcohol use was grouped into “low risk” (<14 drinks per week), “risky” (15-28 drinks per week), and “high risk” (>28 drinks per week). Physical activity was grouped into 200, 200 to 800, >800 moderate activity minutes per week. Usual eating habits over the past 12 months were asked in the ALSWH surveys quantified in daily amounts with pictures to guide assessment of quantities. Items were very detailed with over 74 items of specific food types questioned. The surveys are freely available on the ALSWH website.^29^ Five diet scores were calculated. The DASH score^15^ focused on healthy fat-free foods and reducing sugar. It is recommended as a blood pressure diet being one of U.S. News & World Report's Best Diets of 2025, developed by the National Heart, Lung and Blood Institute. The Mediterranean diet^16^ focused on plant-based foods, healthy fats, and moderate amounts of fish, poultry, and dairy, while limiting red meat and processed foods. The UPF diet score^17^ was calculated based on the proportion of ultra-processed food in the diet which are high in fat and sugar. The DGI^18^ and the ARFS^19^ assess adherence to Australian recommended food groups, including vegetables, fruits, grains, and protein sources, and also assesses intake of less healthy components like saturated fat and added sugar. All scores were calculated and divided in quintiles or tertiles or dichotomized to reduce low counts. For DGI, we were unable to calculate the proportion of whole-meal/whole-grain bread relative to total bread, nor the proportion of lean meats and alternatives relative to total meats, as these relative proportions were not collected in the surveys. Information on type of milk usually consumed was also not available. As the full DGI is out of 150, the reduced version used in this study was scored was out of 120. Diet questions were asked at survey 3 (2003 when aged 25-30 years) and survey 5 (2009 when aged 31-36 years), whereas BMI, physical activity, HDP, smoking, breastfeeding duration, and number of pregnancies were asked at every survey. Glycemic index ranks foods based on the blood glucose response to a given amount of carbohydrate from that food. Glycemic index values of individual food items were obtained from the 2002 International table of glycemic index and glycemic load values.^30^ Dietary glycemic load is the sum of the product of carbohydrate intake from each food by the glycemic index for that food. Glycemic load was divided by total carbohydrate intake to obtain dietary glycemic index.^30^

### Statistical analysis

To estimate the association of HDP with a pCV event, a Kaplan-Meier curve was presented with the unadjusted HRs of a pCV event with HDP. Time to a pCV event began at the birth of their first child. Women were censored at the latest of the end of the linkage data or at their last completed survey as both had information on any major CV events. To determine the final adjusted model a parsimonious Cox proportional hazards model was built for time to a pCV event using a backwards selection method. All covariates of interest were converted to time varying covariates as they were asked at various times across the survey period of 17 years and hence their status may have changed across time. When the status of a covariate was unknown such as they may have been pregnant before survey 3 when the diet questions were asked or they may have been pregnant before the start the ALSWH surveys, the covariate results were back filled as the best representative information. The time varying covariate of HDP was then added to the final model to estimate an adjusted HR (aHR) for the association of pCV events with HDP. Repeat HDP episodes were assessed by adding a time varying count of HDP diagnoses to the unadjusted and the adjusted Cox proportional hazards model, both adjusting for parity. The proportional hazards assumption was tested using visual comparisons and Schoenfeld test with a Bonferroni adjustment for multiple comparisons.

To address the primary aim to identify lifestyle risk factors that modify the high risk of pCV events from HDP, an interaction term of HDP diagnosis and each covariate of interest was added to a cox proportional hazards model of time to a pCV event for any indication that the association of the lifestyle variable acted differently among women with HDP vs those without HDP. *P* values of <0.05 were considered significant for model selection and interaction effects. All statistical analyses were performed with R (version 4·5.0, R Foundation for Statistical Computing).

## Results

There were 10,368 women who had been pregnant in the ALSWH cohort for whom we had an average of 16.7 years of follow-up data up to age 43 to 49 years, with 14.4% (1,493/10,368) having had a diagnosis of HDP. Evidence of HDP was found in the Perinatal Data Collections database which matched the survey data with only an additional 2% found in the hospital and emergency databases for GH and PE, respectively. Our cohort had a median parity of 2, with most mothers (92.3%) born in Australia, the majority educated beyond high school (76.1%) and good representation from outer regional or remote communities (29.2%) (Table 1).

**Table 1:** Baseline Characteristics

Characteristic of Parous Women	HDP	No HDP	Overall (N = 10,368)
Hypertensive disorders of pregnancy (HDP), n (%)	1,493 (100%)	0 (0%)	1,493 (14.4%)
Age at first pregnancy (y), mean (SD)	28.5 (5.4)	29.1 (5.6)	29.0 (5.6)
Country of birth (missing = 71), n (%)			
Australia	1,374 (92.7%)	7,655 (92.2%)	9,504 (92.3%)
Other English country	67 (4.5%)	300 (3.6%)	387 (3.8%)
Europe	15 (1.0%)	80 (1.0%)	99 (1.0%)
Asia	16 (1.1%)	185 (2.2%)	213 (2.1%)
Other	11 (0.7%)	80 (1.0%)	94 (0.9%)
Remoteness, most remote ever, n (%)			
Major city	541 (36.2%)	3,104 (37.1%)	3,825 (36.9%)
Inner regional	523 (35.0%)	2,800 (33.5%)	3,512 (33.9%)
Outer regional/remote	429 (28.7%)	2,453 (29.4%)	3,031 (29.2%)
Marital status, n (%)			
Ever married	1,158 (77.6%)	6,214 (74.4%)	7,669 (74.0%)
Ever divorced/separated/widowed	292 (19.6%)	1,536 (18.4%)	1,887 (18.2%)
Highest education (missing = 8), n (%)			
University	597 (40.0%)	3,856 (46.2%)	4,536 (43.8%)
Certificate/diploma	533 (35.7%)	2,642 (31.6%)	3,344 (32.3%)
Year 12 or equivalent	220 (14.7%)	1,078 (12.9%)	1,448 (14.0%)
Less than year 12	143 (9.6%)	773 (9.3%)	1,032 (10.0%)
Body mass index (BMI) (highest) (missing = 487), n (%)			
Underweight (<18.5 kg/m^2^)	17 (1.2%)	160 (2.0%)	198 (2.0%)
Acceptable (≥18.5 to <25 kg/m^2^)	305 (21.4%)	3,125 (39.1%)	3,630 (36.7%)
Overweight (≥25 to <30 kg/m^2^)	397 (27.8%)	2,367 (29.6%)	2,894 (29.3%)
Obese (≥30 kg/m^2^)	707 (49.6%)	2,336 (29.2%)	3,159 (32.0%)
Ever smoked, n (%)	820 (54.9%)	4,540 (54.3%)	5,705 (55%)
Ever regularly drank alcohol, n (%)	1,218 (81.6%)	6,997 (83.7%)	8,585 (82.8%)
Physical activity (>150 min/week, ever), n (%)	1,391 (93.2%)	7,618 (91.2%)	9,447 (91.1%)
Difficulty sleeping (missing = 13) (worst ever), n (%)			
Never	1,171 (78.5%)	6,537 (78.3%)	841 (8.1%)
Rarely	184 (12.3%)	1,045 (12.5%)	1,682 (16.2%)
Sometimes	115 (7.7%)	626 (7.5%)	3,995 (38.6%)
Often	21 (1.4%)	139 (1.7%)	3,837 (37.1%)
Parity, median (IQR)	2.0 (2.0, 3.0)	2.0 (2.0, 3.0)	2.0 (2.0,3.0)
Breastfeeding ever, n (%)	1,093 (73.2%)	6,188 (74.0%)	7,287 (70.3%)
Breastfeeding average number of months per birth, median (IQR)	6.2 (2.0, 11.5)	8.0 (3.5, 12.0)	8.0 (3.1, 12.0)
Low ultra-processed food diet adherence score at survey 3 (missing = 3,011), n (%)			
Score ≥ 35.5 (worst)	284 (25.8%)	1,357 (22.9%)	1,714 (23.3%)
29.3<score ≤ 35.5	219 (19.9%)	1,308 (22.1%)	1,594 (21.7%)
24.3<score ≤ 29.3	217 (19.7%)	1,194 (20.2%)	1,484 (20.2%0)
19.5<score ≤ 24.9	201 (18.3%)	1,096 (18.5%)	1,358 (18.5%)
Score ≤ 19.5 (best)	178 (16.2%)	967 (16.3%)	1,207 (16.4%)
Mediterranean diet adherence score at survey 3 (missing = 3,011), n (%)			
Score ≤ 5 (worst)	338 (30.8%)	1,636 (27.6%)	2,089 (28.4%)
5<score ≤ 6	223 (20.3%)	1,211 (20.4%)	1,505 (20.5%)
6<score ≤ 7	217 (19.7%)	1,245 (21.0%)	1,529 (20.8%)
7<score ≤ 8	165 (15.0%)	959 (16.2%)	1,165 (15.8%)
Score>8 (best)	156 (14.2%)	871 (14.7%)	1,069 (14.5%)
Dietary Approaches to Stop Hypertension (DASH) diet adherence score at survey 3 (missing = 3,011), n (%)			
Score<21 (worst)	282 (25.7%)	1,216 (20.5%)	1,605 (21.8%)
21 ≤ score<24	271 (24.7%)	1,465 (24.7%)	1,835 (24.9%)
24 ≤ score<26	183 (16.7%)	1,053 (17.8%)	1,291 (17.5%)
26 ≤ score<29	221 (20.1%)	1,223 (20.7%)	1,495 (20.3%)
29 ≤ score (best)	142 (12.9%)	965 (16.3%)	1,131 (15.4%)
Australian Recommended Food Score (ARFS) diet adherence score at survey 3 (missing = 3,011), n (%)			
Score ≤24 (worst)	310 (28.2%)	1,384 (23.4%)	1,801 (24.5%)
24<score ≤30	246 (22.4%)	1,467 (24.8%)	1,798 (24.4%)
30<score ≤34	203 (18.5%)	1,001 (16.9%)	1,258 (17.1%)
34<score ≤40	187 (17.0%)	1,206 (20.4%)	1,444 (19.6%)
Score >40 (best)	153 (13.9%)	864 (14.6%)	1,056 (14.4%)
Australian Dietary Guideline Index (DGI) diet adherence score at survey 3 (missing = 3,011), n (%)			
Score ≤65 (worst)	278 (25.3%)	1,414 (23.9%)	1,800 (24.5%)
65<score ≤70	232 (21.1%)	1,199 (20.2%)	1,500 (20.4%)
70<score ≤75	229 (20.8%)	1,164 (19.7%)	1,459 (19.8%)
75<score ≤80	210 (19.1%)	1,173 (19.8%)	1,433 (19.5%)
Score >80 (best)	150 (13.6%)	972 (16.4%)	1,165 (15.8%)
Energy kJ/day at survey 3 (missing = 3,011), n (%)			
Energy ≤5,580 kJ	350 (23.4%)	1,920 (23.0%)	2,361 (32.1%)
5,580 kJ < Energy ≤7,540 kJ	335 (22.4%)	1,957 (23.4%)	2,409 (32.7%)
Energy >7,540 kJ	414 (27.7%)	2,045 (24.5%)	2,587 (35.2%)
Carbohydrate (g/day) at survey 3 (missing = 3,011), n (%)			
Carbohydrates ≤142.5 g	325 (29.6%)	1,769 (29.9%)	2,185 (28.7%)
142.5 g < carbohydrates ≤195.0 g	371 (33.8%)	1,979 (33.4%)	2,469 (33.6%)
Carbohydrates >195.0 g	403 (36.7%)	2,174 (36.7%)	2,703 (36.7%)
Total fat (g/day) at survey 3 (missing = 3,011), n (%)			
Total fat ≤53.0 g	373 (33.9%)	2,037 (34.4%)	2,500 (34.0%)
53.0 g < total fat ≤76.0 g	314 (28.6%)	1,944 (32.8%)	2,365 (32.1%)
Total fat >76.0 g	412 (37.5%)	1,941 (32.8%)	2,492 (33.9%)
Polyunsaturated fat (g/day) at survey 3 (missing = 3,011), n (%)			
Polyunsaturated fat ≤6.9 g	360 (32.8%)	1,986 (33.5%)	2,441 (33.2%)
6.9 g < polyunsaturated fat ≤10.6 g	350 (31.8%)	1,899 (32.1%)	2,370 (32.2%)
Polyunsaturated fat >10.6 g	389 (35.4%)	2,037 (34.4%)	2,546 (34.6%)
Saturated fat (g/day) at survey 3 (missing = 3,011), n (%)			
Saturated fat ≤21.4 g	373 (33.9%)	2,030 (34.3%)	2,483 (33.8%)
21.4 g < saturated fat ≤31.9 g	312 (28.4%)	1,952 (33.0%)	2,379 (32.3%)
Saturated fat>31.9	414 (37.7%)	1,940 (32.8%)	2,495 (33.9%)
Protein (g/day) at survey 3, (missing = 3,011), n (%)			
Protein ≤66.5 g	355 (32.3%)	2,043 (34.5%)	2,501 (34.0%)
66.5 g < total fat ≤90.0 g	369 (33.6%)	1,918 (32.4%)	2,396 (32.6%)
Total fat >90.0 g	375 (34.1%)	1,961 (33.1%)	2,460 (33.4%)
Fiber (g/day) at survey 3, (missing = 3,011), n (%)			
Fiber ≤15.1 g	382 (34.8%)	2,027 (34.2%)	2,534 (34.4%)
15.1 g < fiber ≤20.9 g	378 (34.4%)	1,976 (33.4%)	2,466 (33.5%)
Fiber >20.9 g	339 (30.8%)	1,919 (32.4%)	2,357 (32.0%)
Sugar (g/day) at survey 3, (missing = 3,011), n (%)			
Sugar ≤63.3 g	337 (30.7%)	1,898 (32.0%)	2,347 (31.9%)
63.3 g < sugar ≤88.8 g	365 (33.2%)	1,947 (32.9%)	2,410 (32.8%)
Sugar >88.8 g	397 (36.1%)	2,077 (35.1%)	2,600 (35.3%)
Glycemic index at survey 3, (missing = 3,011), n (%)			
Glycemic index ≤50.1	235 (21.4%)	1,398 (23.6%)	1,672 (22.7%)
50.1<glycemic index ≤53.7	366 (33.3%)	1,987 (33.6%)	2,451 (33.3%)
Glycemic index >53.7	498 (45.3%)	2,537 (42.8%)	3,234 (44.0%)
Glycemic load at survey 3, (missing = 3,011), n (%)			
Glycemic load ≤73.3	303 (27.6%)	1,648 (27.8%)	2,032 (27.6%)
73.3<glycemic load ≤102.0	369 (33.6%)	1,990 (33.6%)	2,479 (33.7%)
Glycemic load >102.0	427 (38.9%)	2,284 (38.6%)	2,846 (38.7%)

In this cohort 4.3% (448/10,310) had a pCV event, with 58 removed from this estimate due to 50 having a pCV event before the birth of their first child and 8 had no further data after the birth of their first child. pCV events included 28.6% (128/448) ischemic heart disease, stroke 10.9% (49/448), and arrhythmias 23.4% (105/448); see Supplemental Table 1 for detailed breakdown. Women had a mean pregnancy age of 28.8 years, and for those who had a pCV event, it occurred an average of 12.2 years after their first birth.

### Factors associated with pCV events

**Figure 1:**
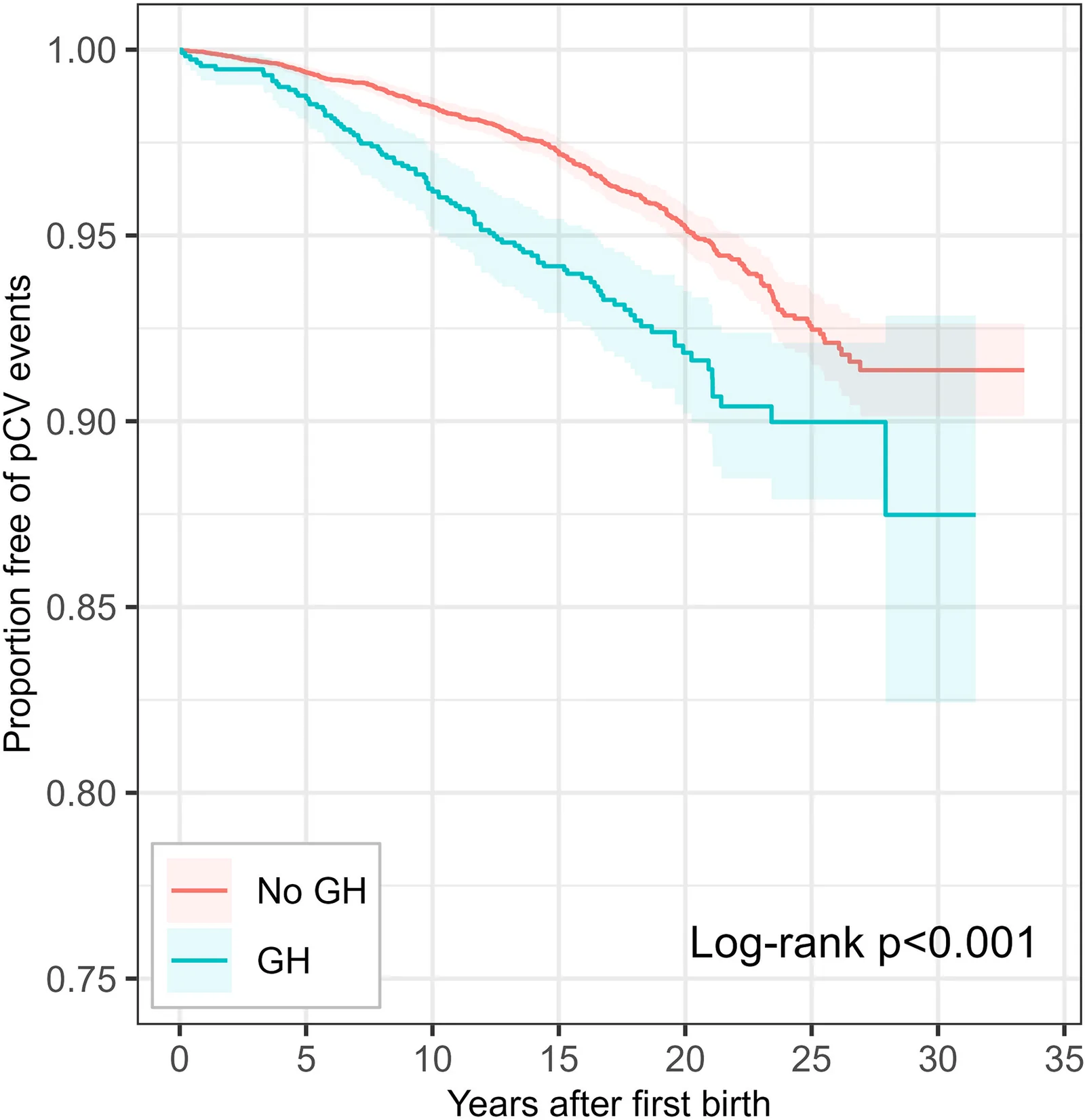
Kaplan-Meier Curve of Time to a pCV Event by Hypertensive Disorders of Pregnancy Compares the proportion event free of a pCV event across time between those with a HDP diagnosis and those without. GH = gestational hypertension; pCV = premature cardiovascular.

In the univariate analysis of parous women under 50 years, obesity, high risk alcohol use, and high intake of saturated fat was associated with a higher risk of pCV, whereas higher levels of physical activity, longer breastfeeding duration, and better adherence to the DGI diet were protective (Table 2). Not smoking, mid-range energy intake, low intake of polyunsaturated fats, mid-range protein intake, and a better education showed a signal of being protective for pCV but were not statistically significant. Sleep, parity, a low UPF diet, Mediterranean diet, DASH diet, ARFS diet, carbohydrates, total fat, fiber, sugar, glycemic index and glycemic load, and socio-economic variables were associated not with pCV. HDP was associated with pCV events in the univariate model (HR: 1.73; 95% CI: 1.39-2.17) and the risk increased with repeat HDP diagnoses (Table 2, Figure 1).

**Table 2:** Lifestyle Risk Factors and Premature Cardiovascular Disease: Univariable Analysis

	HR (95% CI)	*P* Value
Hypertensive disorders of pregnancy (HDP)	1.73 (1.39-2.17)	<0.001
HDP count^a^		<0.001
0	Reference	
1	1.62 (1.26-2.08)	
2+	2.25 (1.48-3.44)	
Country of birth		0.313
Australian born	Reference	
Other English-speaking	0.65 (0.36-1.19)	
Other	1.07 (0.66-1.74)	
Highest education		0.063
Less than year 12	Reference	
Year 12 or equivalent	1.11 (0.85-1.45)	
Certificate/diploma	0.84 (0.64-1.09)	
University	0.81 (0.62-1.05)	
Remoteness		0.096
Major city	Reference	
Inner regional	1.20 (0.97-1.48)	
Outer regional/Remote	0.92 (0.71-1.19)	
Body mass index (BMI)		<0.001
Underweight	0.95 (0.55-1.64)	
Acceptable	Reference	
Overweight	0.99 (0.76-1.27)	
Obese	1.71 (1.36-2.14)	
Smoking status		0.081
Nonsmoker	Reference	
Ex-smoker	1.20 (0.96-1.50)	
Current smoker	1.27 (1.01-1.60)	
Alcohol		0.018
None or rarely	Reference	
Low risk drinker	0.81 (0.67-0.98)	
High risk drinker	1.29 (0.89-1.87)	
Physical activity		<0.001
Physical activity ≤200	Reference	
200 < physical activity ≤799	0.82 (0.66-1.01)	
Physical activity >799	0.63 (0.49-0.80)	
Frequency of poor sleep		0.239
Never	Reference	
Rarely	1.00 (0.78-1.29)	
Sometimes	1.01 (0.79-1.28)	
Often	1.31 (0.99-1.72)	
Parity (per child)	0.99 (0.90-1.09)	0.826
Breastfeeding duration (per month)	0.98 (0.97-0.99)	<0.001
Ultra-processed food diet adherence score		0.358
Score >35.5 (less adherent)	0.87 (0.62-1.22)	
29.3<score ≤35.5	0.93 (0.67-1.29)	
24.3<score ≤29.3	0.77 (0.55-1.09)	
19.5<score ≤24.9	0.73 (0.51-1.03)	
Score ≤19.5 (more adherent)		
Mediterranean diet adherence score		0.324
Score ≤5 (less adherent)	Reference	
5<score ≤6	0.76 (0.56-1.03)	
6<score ≤7	0.96 (0.72-1.29)	
7<score ≤8	0.79 (0.55-1.12)	
Score >8 (more adherent)	0.80 (0.56-1.16)	
Dietary Approaches to Stop Hypertension (DASH) diet adherence score		0.358
Score <21 (less adherent)	Reference	
21 ≤ score<24	1.17 (0.88-1.56)	
24 ≤ score<26	0.98 (0.70-1.38)	
26 ≤ score<29	0.98 (0.71-1.37)	
29 ≤ score (more adherent)	0.78 (0.52-1.18)	
Australian Recommended Food Score (ARFS) diet adherence score		0.790
Score ≤24 (less adherent)	Reference	
24<score ≤30	1.00 (0.73-1.38)	
30<score ≤34	0.92 (0.65-1.31)	
34<score ≤40	0.95 (0.69-1.31)	
Score >40 (more adherent)	0.82 (0.58-1.17)	
Australian Dietary Guideline Index (DGI) diet adherence score		0.021
Score ≤65 (less adherent)	Reference	
65<score ≤70	0.82 (0.61-1.09)	
70<score ≤75	0.67 (0.47-0.95)	
75<score ≤80	0.87 (0.66-1.14)	
Score >80 (more adherent)	N/A	
Energy (kJ/day)		0.060
Energy ≤5,580 kJ	Reference	
5,580 kJ < energy ≤7,540 kJ	0.82 (0.62-1.09)	
Energy >7,540 kJ	1.13 (0.87-1.46)	
Carbohydrates (g/day)		0.540
Carbohydrates ≤142.5 g	Reference	
142.5 g < carbohydrates ≤195.0 g	1.03 (0.78-1.35)	
Carbohydrates >195.0 g	1.15 (0.88-1.49)	
Total fat (g/day)		0.208
Total fat ≤53.0 g	Reference	
53.0 g < total fat ≤76.0 g	1.06 (0.80-1.42)	
Total fat >76.0 g	1.25 (0.95-1.65)	
Polyunsaturated fat (g/day)		0.078
Polyunsaturated fat ≤6.9	Reference	
6.9 g < polyunsaturated fat ≤10.6 g	1.35 (1.02-1.78)	
Polyunsaturated fat >10.6 g	1.29 (0.97-1.70)	
Saturated fat (g/day)		0.036
Saturated fat ≤21.4 g	Reference	
21.4 g < saturated fat ≤31.9 g	0.93 (0.69-1.25)	
Saturated fat >31.9 g	1.27 (0.97-1.66)	
Protein (g/day)		0.083
Protein ≤66.5 g	Reference	
66.5 g < total fat ≤90.0 g	0.77 (0.58-1.02)	
Total fat >90.0 g	1.01 (0.78-1.30)	
Fiber (g/day)		0.583
Fiber ≤15.1 g	Reference	
15.1 g < fiber ≤20.9 g	1.12 (0.86-1.46)	
Fiber >20.9 g	0.99 (0.76-1.29)	
Sugar (g/day)		0.841
Sugar ≤63.3 g	Reference	
63.3<sugar ≤88.8 g	0.96 (0.74-1.26)	
Suga>88.8 g	1.04 (0.80-1.35)	
Glycemic index		0.114
Glycemic index ≤50.1	Reference	
50.1<glycemic index ≤53.7	1.02 (0.77-1.35)	
Glycemic inde>53.7	1.27 (0.98-1.65)	
Glycemic load		0.448
Glycemic load ≤73.3	Reference	
73.3<glycemic load ≤102.0	1.07 (0.81-1.41)	
Glycemic load >102.0	1.18 (0.91-1.54)	

N/A = not available.

a Adjusted for parity.

In the multivariable model, HDP, obesity, and high saturated fat remained significantly associated with a higher risk of pCV, whereas breastfeeding and high protein intake was associated with a lower risk of pCV. Other healthy diet measures, physical activity, and smoking were not associated with pCV after accounting for other covariates (Table 3). HDP diagnosis was associated with pCV in the multivariable model (aHR: 1.68; 95% CI: 1.24-2.28) and for women with repeat HDP the risk was increased (aHR: 2.07; 95% CI: 1.21-3.53) (Table 3).

**Table 3:** Factors Associated With Premature Cardiovascular Disease: Multivariable Model

Covariates in Final Model	aHR (95% CI)	*P* Value
Hypertensive disorders of pregnancy (HDP)	1.74 (1.32, 2.31)	<0.001
Body mass index (BMI)		0.005
Acceptable (reference)	1.00	
Underweight	0.95 (0.42, 2.16)	
Overweight	0.96 (0.70, 1.33)	
Obese	1.60 (1.19, 2.15)	
Breastfeeding (months)		0.023
≤6.5 months (reference)	1.00	
6.5 months < breastfeeding ≤16 months	0.85 (0.64, 1.13)	
>16 months	0.66 (0.49, 0.89)	
Protein(g/day)		0.016
Protein ≤66.5 g (reference)	1.00	
66.5 g < Protein ≤90.0 g	0.60 (0.42, 0.85)	
Protein>90.0 g	0.65 (0.43, 0.96)	
Saturated fat (g/day)		0.038
Saturated fat ≤21.4 g (reference)	1.00	
21.4 g < saturated fat ≤31.9 g	1.20 (0.84, 1.72)	
Saturated fat >31.9 g	1.67 (1.11, 2.53)	
Count of HDP		<0.001
0 (reference)	1.00	
1	1.68 (1.24, 2.28)	
2+	2.07 (1.21, 3.53)	
Parity		0.253
1 (reference)	1.00	
2+	0.82 (0.58,1.15)	
Body mass index (BMI)		0.006
Acceptable (reference)	1.00	
Underweight	0.94 (0.41, 2.14)	
Overweight	0.96 (0.70, 1.32)	
Obese	1.59 (1.18, 2.13)	
Breastfeeding (months)		0.058
≤6.5 months (reference)	1.00	
6.5 months < breastfeeding ≤16 months	0.87 (0.65, 1.16)	
>16 months	0.69 (0.50, 0.94)	
Protein (g/day)		0.017
Protein ≤ 66.5 g (reference)	1.00	
66.5 g < Protein ≤90.0 g	0.60 (0.43, 0.86)	
Protein >90.0 g	0.65 (0.44, 0.96)	
Saturated fat(g/day)		0.038
Saturated fat ≤21.4 g (reference)	1.00	
21.4 g < saturated fat ≤31.9 g	1.21 (0.85, 1.72)	
Saturated fat >31.9 g	1.68 (1.11, 2.53)	

aHR = adjusted HR; pCV = premature cardiovascular.

### The effect of lifestyle modifying the risk of HDP on pCV events

**Figure 2:**
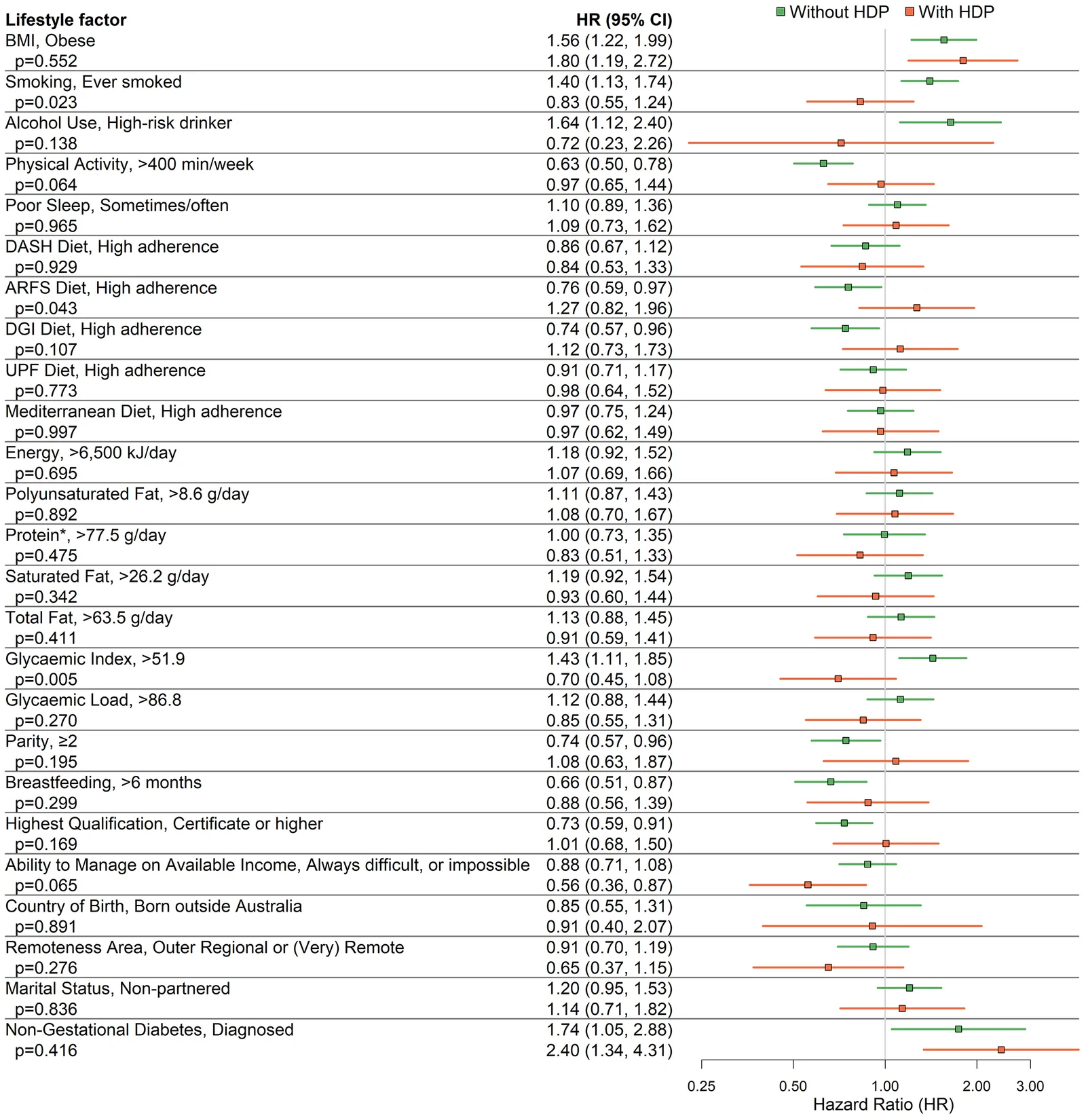
Risk of Premature Cardiovascular From Lifestyle Risk Factor by HDP Status Compares the risk of each lifestyle risk factor of a pCV event between those with a HDP diagnosis and those without. ∗Adjusted for saturated fat. ARFS = Australian Recommended Food Score; BMI = body mass index; DASH = Dietary Approaches to Stop Hypertension; DGI = Australian Dietary Guideline Index; HDP = hypertensive disorders of pregnancy; UPF = Ultra-Processed Food.

In women with HDP compared to women without HDP, there was heterogeneity in the association (significant *P* value for interaction) of several lifestyle risk factors: smoking, the ARFS diet, and glycemic index. Thus, smoking was associated with an increased risk of pCV events in women without HDP, but there was no association in women with HDP (*P* interaction = 0.023) (Figure 2). Adherence to ARFS was associated with a lower risk of pCV events in women without HDP, and no association in women with HDP (*P*-interaction = 0.043). High glycemic index was associated with higher risk in women without HDP and no association in women with HDP (*P* interaction = 0.005). There was no statistically significant heterogeneity in association found for other lifestyle factors of physical activity, protein, fat intake, and obesity. Details are in Figure 2. Overall results are demonstrated in the Central Illustration. We found that the proportional hazards assumption was not violated, with the time varying effect of each covariate visually constant and not statistically significant.

**Central Illustration:**
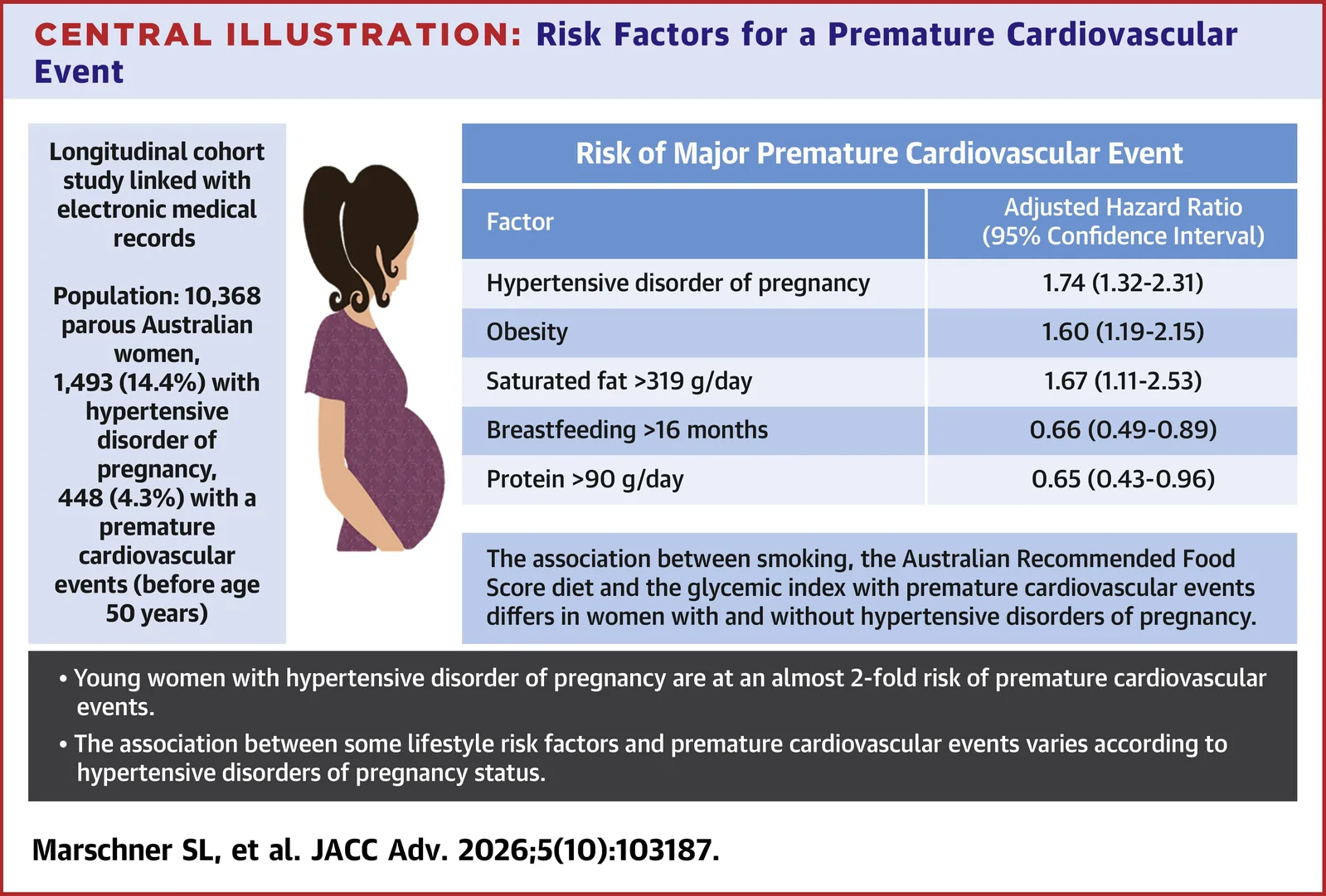
Risk Factors for a Premature Cardiovascular Event Identifies predictors of premature cardiovascular events and estimates the risk associated with HDP and whether any lifestyle behaviors can mitigate this risk.

## Discussion

This study found further evidence that HDP is associated with pCV events for women under 50 years and that this risk for a pCV event is close to 2-fold after accounting for covariates. Obesity and higher saturated fat intake were associated with higher risk of pCV events, whereas breastfeeding and higher protein intake were associated with a lower risk of pCV events. The association between smoking, the Australian Recommended Food Score diet, and glycemic index with pCV events differed in women with and without HDP.

It is well accepted that lifestyle risk factor modification reduces CV risk.^31^ Most of this evidence is from cohort studies and trials in older patient populations and associated with all CV events. There is less research that focuses on young populations and pCV events. A cohort study with a primary outcome of coronary heart disease (CHD), rather than our broader outcome of major CVD outcomes, used the Nurses’ Health Study II of almost 90,000 women, and found evidence that nonsmoking, a healthy BMI, exercise, and a healthy diet were independently and significantly associated with lower CHD risk.^32^ The women in this study were ages 27 to 44 years at baseline in 1991 and followed for 20 years until 2011. Despite reporting a mean age of CHD of 50.3 years, many CHD diagnoses would have been later in life.

Previous studies have consistently demonstrated heart-healthy diets to be associated with reduced CV events. In a large cohort study and meta-analysis, the DASH diet was associated with reduced CVD death in women and reduced CVD and CHD events.^33^^,^^34^ Similarly ultraprocessed foods was associated with increased CVD death in a large cohort study^35^ and in meta-analysis ultraprocessed foods was shown to be associated with a range of CV events (CVD death, myocardial infarction, stroke, transient ischemic attack, coronary intervention, stent thrombosis, peripheral vascular intervention, hospitalization for unstable angina, and acute heart failure).^36^ The PREDIMED trial was a randomized clinical trial that demonstrated the protective effect of the Mediterranean diet on a composite outcome of myocardial infarction, stroke, and CVD death.^37^ However, associations of heart-healthy diets with pCV were not clearly observed in the current ALSWH analyses. Although noting a weak association for glycemic index and ARFS diets among those without HDP, this was not the case among people with HDP. This observation may be related to the focus on pCV events, the broader definition used for all pCV events in this study, or the use of simplified versions of the diet scores—which were simplified due to missing information on whole-grain bread, lean meat proportions, and milk type. Our study did find, however, 2 important dietary findings: high saturated fat intake was associated with higher risk of pCV events, whereas high protein intake was associated with lower risk of pCV events. There has been debate as to whether a high protein diet increases CVD risk.^38^

There is growing evidence that breastfeeding is protective for women.^39^ The 45 and Up cohort study of over 100,000 parous Australian women using linked data found breastfeeding is protective against CVD mortality with an estimated risk reduction of 34%.^40^ A recent Nurses’ Health Study I and II with almost 20,000 women with gestational diabetes or type 2 diabetes found longer duration of breastfeeding is associated with lower risk of CVD.^41^ None of these studies focus on pCV events. Our study added to this evidence finding that duration of breastfeeding was an important independent protective factor for pCV events. Our study also demonstrated that this protection was consistent for women with or without a HDP diagnosis. Previous studies have also shown higher parity (≥5 vs 2 children) to be associated with an increased risk of CVD;^42^ however, our study found no evidence of parity being a predictor for pCV events, although the median parity of our cohort was 2.

### Study limitations

Hospital records and the ALSWH surveys do not include important traditional CV risk factors for ischemic disease such as lipid information and family history of CV which could be important confounders. This data have some limitation due to the self-reported survey data which makes measuring lifestyle factors such as diet, exercise, and alcohol less reliable. Diet is particularly less reliable using surveys but is inherently difficult to measure in any form. Given that only 14% of the cohort had a HDP diagnosis and 4% had a pCV event, this study is likely to be underpowered to detect interaction effects. This study has the advantage of using of time varying covariates utilizing frequent surveying every 3 years, so the results have comparisons within a participant across time and between participants. In addition, the richness of the data means that many covariates can be assessed and compared within the same cohort. This study was for women born in Australia between 1973 and 1978 and important to note lifestyles and health care would reflect these times. We were unable to separate GH, PE, and E. These conditions differ in severity and may differ in their association with pCV events. Residual confounding may affect the results and can occur if some aspects of diet are insufficiently captured. The results must be interpreted with caution.

## Conclusions

This study provides further evidence that HDP is associated with an increased risk of pCV. For women with repeat occurrences of HDP, the risk of pCV is higher than those with 1 episode of HDP. In all women, obesity and high saturated fat diet were associated with higher pCV rates, whereas breastfeeding and high protein diet were associated with lower pCV rates, suggesting that preventative lifestyle measures should be encouraged in young women. Future studies with longer follow-up are required to better understand the longer-term impact of these lifestyle variables.

Perspectives **COMPETENCY IN MEDICAL KNOWLEDGE:** Women who have had a hypertensive disorder of pregnancy are at close to twice the risk of a premature cardiovascular event. Breastfeeding, low saturated fat, high protein diets and weight management are associated with a reduce the risk of premature cardiovascular events.

**TRANSLATIONAL OUTLOOK:** Women with a history of hypertensive disorder of pregnancy need strong prevention management to prevent premature cardiovascular events.

## Funding support and author disclosures

Dr Chow is supported by a National Health and Medical Research Investigator Grant APP2043452. All other authors have reported that they have no relationships relevant to the contents of this paper to disclose.
