# Supplementary material for: Association Between Hypertensive Disorders of Pregnancy, Lifestyle Factors, and Premature Cardiovascular Events
Source: JACC Adv. 2026 Aug 31;5(10):103187. doi: 10.1016/j.jacadv.2026.103187 (PMC13562067; doi:10.1016/j.jacadv.2026.103187)
Supplement: Supplementary file 1 [file mmc1.docx]

**Appendix Table 1:** Details of pCV events

|  | pCV events,  n (%) N=448* | Ischaemic secondary event,  n (%) N=240* |
| --- | --- | --- |
| **I11-I13 (hypertensive heart diseases)** |  |  |
| I11 Hypertensive heart disease | 1 (0.2%) | 1 (0.4%) |
| I12 Hypertensive kidney disease | 0 | 0 |
| I13 Hypertensive heart and kidney disease | 0 | 0 |
| **I20-I25 Ischaemic heart disease** |  |  |
| I20 Angina pectoris | 79 (17.6%) | 84 (35.0%) |
| I21 Acute myocardial infarction | 10 (2.2%) | 10 (4.2%) |
| I23 Certain current complications following acute myocardial infarction | 0 | 0 |
| I24 Other acute ischaemic heart diseases | 3 (0.7%) | 3 (1.3%) |
| I25 Chronic ischaemic heart disease | 0 | 0 |
| Ischaemic heart disease via ALSWH CCMS | 36 (8.0%) | 38 (15.8%) |
| **I26-I28 Pulmonary heart disease and diseases of pulmonary circulation** |  |  |
| I26 Pulmonary embolism | 53 (11.8%) | N/A |
| I27 Other pulmonary heart diseases | 1 (0.2%) | N/A |
| I28 Other diseases of pulmonary vessels | 0 | N/A |
| **I34-36, I42, I44, I46-I51 (selected other forms of heart disease)** |  |  |
| I34 Nonrheumatic mitral valve disorders | 0 | N/A |
| I35 Nonrheumatic aortic valve disorders | 1 (0.2%) | N/A |
| I36 Nonrheumatic tricuspid valve disorders | 0 | N/A |
| I42 Cardiomyopathy | 2 (0.4%) | 2 (0.8%) |
| I44 Atrioventricular and left bundle-branch block | 1 (0.2%) | N/A |
| I46 Cardiac arrest | 4 (0.9%) | 4 (1.7%) |
| I47 Paroxysmal tachycardia | 41 (9.2%) | N/A |
| I48 Atrial fibrillation and flutter | 18 (4.0%) | N/A |
| I49 Other cardiac arrhythmias | 46 (10.3%) | N/A |
| I50 Heart failure | 2 (0.4%) | 4 (1.7%) |
| I51 Complications and ill-defined descriptions of heart disease | 5 (1.1%) | N/A |
| **I61-I67, I69 (selected cerebrovascular disease)** |  |  |
| I61 Intracerebral haemorrhage | 5 (1.1%) | 6 (2.5%) |
| I62 Other nontraumatic intracranial haemorrhage | 4 (0.9%) | 5 (2.1%) |
| I63 Cerebral infarction | 6 (1.3%) | 7 (2.9%) |
| I64 Stroke, not specified as haemorrhage or infarction | 10 (2.2%) | 12 (5.0%) |
| I65 Occlusion and stenosis of precerebral arteries, not result cerebral infarction | 0 | 0 |
| I66 Occlusion and stenosis of cerebral arteries, not result cerebral infarction | 0 | 0 |
| I67 Other cerebrovascular diseases | 8 (1.8%) | 10 (4.2%) |
| I69 Sequelae of cerebrovascular disease | 0 | 0 |
| Cerebrovascular disease via ALSWH CCMS | 16 (3.6%) | 18 (7.5%) |
| **I70-I77 (selected diseases of arteries, arterioles and capillaries)** |  |  |
| I70 Atherosclerosis | 3 (0.7%) | 3 (1.3%) |
| I71 Aortic aneurysm and dissection | 1 (0.2%) | 1 (0.4%) |
| I72 Other aneurysm and dissection | 3 (0.7%) | 3 (1.3%) |
| I73 Other peripheral vascular diseases | 1 (0.2%) | 1 (0.4%) |
| I74 Arterial embolism and thrombosis | 5 (1.1%) | 5 (2.1%) |
| I77 Other disorders of arteries and arterioles | 5 (1.1%) | N/A |
| **I80 (selected diseases of veins, lymphatic vessels and lymph nodes, not elsewhere )** |  |  |
| I80 Phlebitis and thrombophlebitis (I80.2 DVT)  (Other than I80.2) | 37 (8.3%)  20 (4.5%) | N/A |
| **G45,G46 (selected episodic and paroxysmal disorders)** |  |  |
| G45 Transient cerebral ischaemic attacks and related syndromes | 21 (4.7%) | 21 (8.8%) |
| G46 Vascular syndromes of brain in cerebrovascular diseases | 0 | 0 |

*The first of each event, which is why the subset of the secondary ischaemic events may be larger than the original pCV event for a particular type of outcome.
